# Cow's Milk and Rickets

**Published:** 1908-05-09

**Authors:** 


### Editor's Letter-Box.

### COW'S MILK AND RICKETS.

Sir,—Rickets from deficient calcium in cow's milk sounds like a false alarm. The proportion of salts in cow's milk to that in human milk is as .7 to .3, the reason being perhaps that the calf has at birth a much more developed osseous system than the human baby. Then Sir A. E. Wright has shown that the addition of a pint of milk—the ordinary commercial article—to a diet will increase blood coagulability surprisingly, owing to the large amount of lime salts it gives up to the blood. Most people now partially decalcify milk for typhoid patients, to lessen risk of thrombosis. In fact, Wright regards milk as acting very like a medicinal preparation of calcium. He also considers that cow's milk often disagrees with babies from its overrichness in these salts, and recommends preliminary decalcification. It is certainly a pretty experiment to add rennet to three portions of milk; one untreated and the other two containing respectively a half and one grain of sodium citrate to the ounce. The finer curd in the two latter is very noticeable. Considerable dilution would, of course, obviate the necessity of this.

I am, yours etc.,

London, May 2. J.

